# *Agrobacterium tumefaciens*-Mediated Transformation of *Candida glabrata*

**DOI:** 10.3390/jof8060596

**Published:** 2022-06-02

**Authors:** Samantha D’Spain, Pilar I. Andrade, Nohelli E. Brockman, Jianmin Fu, Brian L. Wickes

**Affiliations:** The Department of Microbiology, Immunology, and Molecular Genetics, The University of Texas Health Science Center at San Antonio, San Antonio, TX 78229-3900, USA; sd6eh@virginia.edu (S.D.); pilar.andrade@bcm.edu (P.I.A.); brockmann@uthscsa.edu (N.E.B.); fuj@uthscsa.edu (J.F.)

**Keywords:** antifungal, pathogen, gene disruption, yeast

## Abstract

The use of broad-spectrum antimycotic therapy, immunosuppressive therapy, and indwelling medical devices has contributed to the increased frequency of mucosal and systemic infections caused by *Candida glabrata*. A major concern for *C. glabrata* and other *Candida* spp. infections is the increase in drug resistance. To address these issues, additional molecular tools for the study of *C. glabrata* are needed. In this investigation, we developed an *Agrobacterium tumefaciens* transformation system for *C. glabrata*. A number of parameters were investigated to determine their effect on transformation frequency, and then an optimized protocol was developed. The optimal conditions for the transformation of *C. glabrata* were found to be an infection incubation temperature of 26 °C, 0.2 mM acetosyringone in both induction media and co-culture media, 0.7% agar concentration, and a multiplicity of infection of 50:1 *A. tumefaciens* to *C. glabrata*. Importantly, the frequency of multiple integrations was low (5%), demonstrating that *A. tumefaciens* generally integrates at single sites in *C. glabrata*, which is consistent with other fungal *A. tumefaciens* transformation systems. The development of this system in *C. glabrata* adds another tool for the molecular manipulation of this increasingly important fungal pathogen.

## 1. Introduction

Historically, *Candida glabrata* has been considered a commensal organism of low pathogenicity that rarely causes serious infections in humans [1]. However, with the increase in hospitalized patients who are immunosuppressed by disease or treatment, mucosal and systemic infections caused by *C. glabrata* have increased significantly, to the point that nosocomial infections are a serious healthcare concern [2]. A major factor contributing to this problem is the increase in drug resistance. *Candida* species are responsible for more than 8–10% of all nosocomial bloodstream infections, with the most frequently encountered species being *C. albicans* and *C. glabrata* [3,4]. Treatment of these infections has led to a predicted increase in the incidence of antifungal drug resistance. In fact, both species are on the World Health Organization and the Center for Disease Control Antibiotic threat lists [5,6]. Compared to other non-*albicans Candida* (NAC) species, *C. glabrata* is increasingly prevalent in systemic infections and has one of the highest mortality rates of all *Candida* species [7]. Unfortunately, clinicians are faced with limited antifungal choices.

Currently, there are only three categories of antifungals routinely used for the treatment of systemic candidiasis, polyenes, azoles, and echinocandins, all of which show increasing frequencies of resistance [8]. The troubling lag in the development of new antifungals has contributed to treatment problems associated with systemic infections caused by drug-resistant *Candida* strains. The lack of a continuous pipeline of new antifungal drugs has resulted in important clinical consequences that continue to grow and will not be adequately addressed in the near future without new entries into the antifungal market. For example, echinocandins were developed almost 40 years ago but were not FDA-approved until 2001 [9]. However, even with this newest antifungal class, resistance is already a problem. Complicating this situation has been the recent observations of resistance in *C. glabrata* without previous drug exposure [10,11], and cross resistance, where resistance arising due to treatment with one drug can lead to resistance to another structurally unrelated drug that has not been used for treatment [12,13]. The increasing clinical significance of *C. glabrata* requires more investigation, which in turn requires better tools.

Insertional mutagenesis, which can be accomplished using many different methods, has proven to be a powerful tool for identifying new genes and their functions [14]. A widespread insertional mutagenesis method that has had great success in fungi employs a bacterial pathogen of plants called *Agrobacterium tumefaciens*. *Agrobacterium tumefaciens* transformation has been developed for almost every major human fungal pathogen and is an ideal method for insertional mutagenesis [15]. In contrast to plasmid-based transformation systems, which may require specific delivery systems (electroporation, biolistics, spheroplasting, etc.), *A. tumefaciens* transformation utilizes the same general protocol regardless of the host organism [15]. Transformation occurs by mixing transformation-competent *A. tumefaciens* cells, which carry an engineered plasmid containing a fungus-specific selectable marker, with fungal host cells. The bacterium transfers part of the plasmid into the fungus host cell, which integrates randomly throughout the genome. Fungal transformants can then be selected by growth on agar, which is enabled by the integrated *A. tumefaciens* DNA fragment that carries the selectable marker.

With this knowledge, the development of an optimized system of *A. tumefaciens-*mediated transformation (ATMT) for *C. glabrata* can greatly enhance our understanding of this fungus and potentially lead to the development of better antifungals, as well as enhance our understanding of the pathogenesis of this organism. The generation of large insertion libraries will allow screening for virtually any phenotype that can be detected with an assay. One of the simplest examples is the use of replica plating to identify mutants of interest from the library. These experiments simply require the identification of *A. tumefaciens* conditions that result in a large number of transformants. The goal of this study, therefore, was to develop an *A. tumefaciens* transformation system for *C. glabrata* and then optimize it to maximize transformation yields. 

## 2. Materials and Methods

### 2.1. Strains and Plasmids

*Candida glabrata* wild-type strain, WSA-1572 (CBS138, Centraalbureau voor Schimmelcultures (CBS), Fungal Biodiversity Centre; Utrecht, The Netherlands) was used as the host strain for all transformations. This strain is the type culture of *C. glabrata* and has a sequenced and annotated genome. *Agrobacterium* *tumefaciens* strain EHA105 [16] was the host bacterium for plasmid p624, which was used as the transforming plasmid. Plasmid p624 has a *C. albicans* actin promotor driving the NAT (Nourseothricin resistance) gene, a *C. albicans* actin terminator, and contains *A. tumefaciens* elements that assist its transformation and integration into *C. glabrata*, including the right and left border sequences. It was constructed by inserting a 1.9 kb SpeI/SmaI fragment from plasmid pJK850 [17] containing the *NAT1* resistance gene and the *C. albicans* actin (*ACT1*) promoter and terminator sequences into a SpeI/SmaI digest of pDHt-SK [18] to make plasmid p624. pDHt-SK was a gift from K.J. Kwon-Chung. pJK850 was a gift from Julia Kohler.

### 2.2. Media

Yeast strains were kept as frozen stocks at −80 °C in yeast extract, peptone, and dextrose (YPD) broth, composed of 1.0% yeast extract (Sigma-Aldrich, St. Louis, MO, USA), 2.0% peptone (Research Products International, Mt. Prospect, IL, USA), 2.0% dextrose (EM Science, Gibbstown, NJ, USA), and 15% glycerol (Fisher Scientific, Fair Lawn, NJ, USA). Bacterial strains were kept in frozen stocks of Luria Broth (LB), composed of 1.0% yeast extract, 2.0% tryptone (FisherBiotech, Fair Lawn, NJ, USA), 2.0% sodium chloride (EMD Chemicals, Gibbstown, NJ, USA), and 25% glycerol, at −80 °C. Minimal media (MM) [19] was composed of 500 μL K-buffer (pH 7.0), 1.0 mL M-N buffer, 50 μL 1% CaCl_2_·2H_2_O, 500 μL 20% glucose, 50 μL 0.1% FeSO_4_·7H_2_O 250 μL Spore elements, 125 μL 20% NH_4_NO_3_, 100 μg/mL kanamycin (Gold Biotechnology, St. Louis, MO, USA), and dH_2_O to 50 mL. This medium was filter sterilized. Induction media (IM) [19] was composed of 80 μL K-buffer (pH 5.7), 2.0 mL M-N buffer (3.0 g MgSO4·7H_2_O, 1.5 g NaCl, per liter) 100 μL 1% CaCl_2_·2H_2_O, 100 μL 0.1% FeSO_4_·7H_2_O, 500 μL spore elements (100 mg ZnSO_4_·7H_2_O, 100 mg CuSO_4_·5H_2_O, 100 mg H_3_BO_3_, 100 mg MnSO_4_·H_2_O, 100 mg Na_2_MoO_4_, per liter), 250 μL 20% NH_4_NO_3_, 1.0 mL 50% glycerol, 1.0 mL 20% glucose, 4.0 mL MES (pH 5.5), 200 μL 100 mM acetosyringone (Sigma-Aldrich, St. Louis, MO, USA), 100 μg/mL kanamycin, and dH_2_O to 100 mL. This medium was filter sterilized. The co-culture medium was prepared from induction media with 1.5% agar (Becton, Dickinson, Sparks, MD, USA) added for solidification [19], without MES and acetosyringone, which were filter sterilized and added after autoclaving. Each medium was prepared fresh on the day of its use.

### 2.3. Growth Conditions

*A. tumefaciens* and *C. glabrata* were cultured on media plates (LB agar with 100 μg/mL kanamycin for *A. tumefaciens*, YPD agar for *C. glabrata*) at 28 °C and 30 °C, respectively, 48 h prior to transformation. Because *A. tumefaciens* grows optimally at close to room temperature, incubations for *A. tumefaciens* growth and yeast–bacteria co-culture were done in a Mini Low Temperature Cooling Incubator (Fisher Scientific, Pittsburgh, PA, USA). Twenty-four hours prior to transformation, *A. tumefaciens* cells were subcultured into 30 mL of minimal media (MM), and *C. glabrata* cells were subcultured into 3 mL of YPD broth. Both cultures were shaken overnight at 250 RPM at 28 °C.

### 2.4. Standard Transformation Protocol

**Figure 1:**
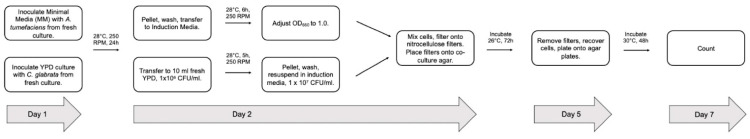
Flowchart of the ATMT protocol.

On the day of transformation, yeast cells were subcultured into 10 mL of fresh YPD broth, and *A. tumefaciens* cells were subcultured into 30 mL of induction media (IM) (Figure 1). The *A. tumefaciens* and *C. glabrata* cultures were shaken for 6 and 5 h, respectively, at 28 °C at 250 RPM. After 5 h, yeast cells were counted with a hemocytometer and harvested when the cell density was approximately 1 × 10^7^ CFU/mL. When the *C. glabrata* cells were ready, they were pelleted, washed twice with 10 mL sterile water, counted again, and then adjusted to 10^8^ cells/mL in induction medium. After 6 h, the optical density (OD_660_ nm) of the *A. tumefaciens* cells was taken and adjusted to 1.0 in induction medium. The 5-h growth period for *C. glabrata* was chosen because we found that at 5 h, by the time we finished counting, diluting, and adjusting, we were at 6 h, which coincided with the *A. tumefaciens* culture being ready. This coordination let us synchronize the mixing step. The adjusted *A. tumefaciens* and *C. glabrata* suspensions, at a 10:1 ratio of *A. tumefaciens* (1 × 10^8^ cells total) to *C. glabrata* (1 × 10^7^ cells total) were mixed and filtered onto 13 mm Nitrocellulose membranes (Millipore, Billerica, MA, USA) using a QiAvac 24 filtration apparatus (Qiagen, Valencia, CA, USA) fitted with 13 mm plastic Swinnex filter holders (Millipore, Billerica, MA, USA). The filters were placed onto 1.5% agar co-cultivation media plates, which were incubated at 26 °C for 72 h. After 72 h, the membranes were removed and suspended in 1 mL of sterile water and vortexed to resuspend cells, which were then plated onto YPD media plates containing 200μg/mL Nourseothricin (Gold Biotechnology, St. Louis, MO, USA) for selection of yeast transformants and 100 μM Cefotaxime (CEFO) (Gold Biotechnology, St. Louis, MO, USA) to kill *A. tumefaciens* cells. The experiments were repeated independently three times with three replicates. Plates were incubated for 48 h at 30 °C, counted, and then analyzed by a paired *t*-test.

### 2.5. Optimization of the Agrobacterium Mediated Transformation System for C. glabrata 

To effectively optimize the ATMT system, several variables were tested to observe the effect on transformation efficiency. Some of the variables tested were based on previous studies showing their importance for transformation efficiency [20,21]. Variables that were tested included temperature, acetosyringone concentration in induction media and/or co-culture media, ratio of *A. tumefaciens* cells to *C. glabrata* cells, a purine synthesis inhibitor (Mizoribine, Sigma-Aldrich, St. Louis, MO, USA), spheroplasting (using Zymolyase, US Biological, Salem, MA, USA), an opine (Nopaline, Toronto Research Chemicals, Toronto, ON, Canada), and agar concentration.

### 2.6. Identification of Insertion Site by Vectorette PCR

The insertion site of the T-DNA in the host genome was identified using vectorette PCR [22,23]. DNA was extracted using the Masterpure Yeast DNA Purification Kit (Lucigen, Inc., Middleton, WI, USA). Transformants were patched onto YPD + NAT + CEFO plates, where they were grown to about 1 cm^2^. The patch was then scraped off the media and suspended in a 2 mL screw tube (Biospec, Inc., Bartlesville, OK, USA) containing 200 μL of Tissue and Cell Lysis Solution, 5 μL of RNase (Sigma-Aldrich), and 100 μL of 0.5 mm glass beads (Biospec, Inc.). The samples were shaken on a mini bead beater (Biospec, Inc.) at the highest setting for 1 min and then incubated in a hot water bath at 65 °C for one hour. The samples were next placed on ice for 5 min, followed by the addition of 100 μL of MPC Protein Precipitation Reagent (Lucigen). Samples were vortexed for 10 s then centrifuged for 10 min at 10,000× *g* to pellet cell debris. The supernatant was transferred to a new 1.5 mL microfuge tube. One milliliter of ethanol was added, and the tubes were mixed thoroughly by inversion. The samples were then incubated overnight at 4 °C. Next, the samples were centrifuged at full speed for 15 min to pellet the DNA. The supernatant was removed, and the pellet was washed with 500 μL of 70% ethanol. The ethanol was removed, and the tubes were dried at room temperature. The DNA was then suspended in 50 μL of sterile Dnase and RNAse-free water (Sigma-Aldrich). DNA was prepared for vectorette PCR by restriction enzyme digestion with HinP1I (New England Biolabs, Ipswich, MA, USA) according to the manufacturer’s instructions. After digestion, the reaction was cleaned using a QIAquick PCR Purification Kit (Qiagen, Inc., Germantown, MD, USA) and then digested DNA was ligated to the vectorette adapters. Adapters were prepared from four microliters of a 10 μM stock concentration of Vect.53 (5′-CTCTCCCTTCTCGAATCGTAACCGTTCGTACGAGAATCGCTGTCCTCTCCTTC-3′) and Vect.57.GC (5′-CGGAAGGAGAGGAAGAGAAGGGAGAG-3′), which were annealed in 11.2 μL of TE, pH 8.0, by heating to 65 °C for 5 min in a thermocycler followed by the addition of 0.8 μL of 50 mM MgCl_2_. The sample was incubated once again at 65 °C for 10 min and then slowly cooled to room temperature by turning off the thermocycler. Vectorette adapters were ligated to digested DNA in a reaction consisting of 2 μL of adapters, 2 μL (10 ng) of DNA, 1 μL T4 ligase (New England Biolabs), 1 μL 10× T4 ligase buffer, and 4 μL H_2_O. The reaction was incubated at 16 °C overnight, then diluted 1:10 and 1:100. Vectorette PCR was performed on 1 μL of the ligation mix with EconoTaq DNA Polymerase (Lucigen) using pDHt R.bor#2.F (5′-TCGTTTCCCGCCTTCAGTTT-3′), which is located within the predicted *A. tumefaciens* insertion sequence close to the border sequence, and the vectorette adapter primer Vect.B21.PCR.Nest (5′-CGTAACCGTTCGTACGAGAAT-3′). PCR conditions consisted of 2 min and 10 s at 94 °C, 40 cycles of 30 s at 94 °C (denaturation), 30 s at 60 °C (annealing), and 30 s at 72 °C (extension), and a final extension of 72 °C for 5 min. The PCR product was then run on a 0.8% agarose gel and stained with ethidium bromide. If the samples were smeared, the next lower dilution was used.

## 3. Results

### 3.1. Effect of Co-Cultivation Parameters on Transformation Efficiency

**Figure 2:**
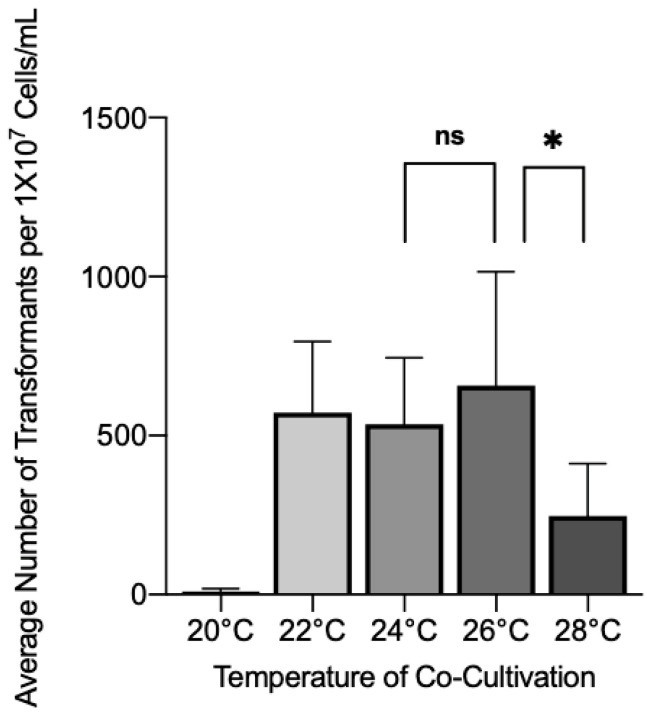
Wild-type *C. glabrata* was co-cultivated with *A. tumefaciens* containing plasmid p624 for 72 h at a variety of temperatures to determine the optimal temperature for transformant yield. Brackets with * above bars indicate groups with statistically significant differences. No significant difference indicated with ns. ns = *p* > 0.05, * = *p* ≤ 0.05.

We first tested the effect of co-cultivation temperature on *A. tumefaciens* transformation efficiency to determine the optimal temperature for infection to occur. Wild-type *C. glabrata* was co-cultivated with *A. tumefaciens* containing plasmid p624 and then incubated at various temperatures for 72 h. After plating on Nourseothricin, Nourseothricin resistant (NAT^r^) colonies were scored. The highest average yield of transformants was found to be at 26 °C with an average of 658 transformants per 1 × 10^7^ cells/mL (Figure 2). There were no significant differences from 22 °C or 24 °C, however, 28 °C and 20 °C showed significant drop-offs in NAT^r^ colonies, with few colonies seen at 20 °C.

### 3.2. Effect of Acetosyringone on Transformation Efficiency

**Figure 3:**
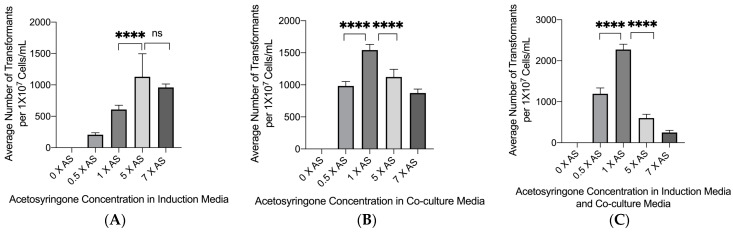
The influence of acetosyringone concentration on transformation frequency. (**A**). *Agrobacterium tumefaciens* strain with plasmid p624 was cultured in induction media with various concentrations of acetosyringone and shaken for 6 h at 28 °C at 250 RPM and then mixed with wild-type *C. glabrata*, which were then placed onto nitrocellulose membranes and plated onto co-culture medium with an acetosyringone concentration of 1 × AS for 72 h at 26 °C. Brackets with **** above bars indicate groups with statistically significant differences. No significant difference indicated with ns. **** = *p* ≤ 0.0001, ns = *p* > 0.05. (**B**). Wild-type *C. glabrata* and *A. tumefaciens* were incubated on co-culture medium with various concentrations of acetosyringone for 72 h at 26 °C (the acetosyringone concentration in induction media was 1 × AS). Brackets with **** above bars indicate groups with statistically significant differences. **** = *p* ≤ 0.0001 (**C**) Combined acetosyringone conditions from (**A**,**B**). Brackets with **** above bars indicate groups with statistically significant differences. **** = *p* ≤ 0.0001.

Acetosyringone is a phenolic compound secreted by plant cells when wounded. This compound induces the virulence genes of *A. tumefaciens* and is a critical component of the ability of *A. tumefaciens* to transfer T-DNA into host cells [24]. Acetosyringone was added in three separate ways during the transformation process. It was added to the induction media only, in which *A. tumefaciens* was sub-cultured prior to plating with *C. glabrata*. It was also added to the co-culture medium only, on which the *A. tumefaciens* and *C. glabrata* mixture was incubated for 72 h at 26 °C. It was also added to both media. Figure 3A shows the results of adding acetosyringone to the induction medium only. There was no significant difference between 0.5× and 5× the standard amount, although both showed slightly reduced numbers of transformants. Increasing the amount from the baseline levels resulted in decreasing numbers of transformants. Adding acetosyringone to the co-culture medium only showed the same type of pattern, although the number of transformants was comparatively less at each concentration than when it was added to the induction medium only (Figure 3B). However, the greatest effect on transformation frequency occurred when acetosyringone was added to both the induction and co-culture media (Figure 3C). In this case, at the optimum concentration, approximately twice the number of transformants were observed compared to adding acetosyringone to the induction media, and four times the number of transformants were observed when acetosyringone was added to the co-culture medium. Interestingly, at the 0 concentration of acetosyringone, no transformants were observed demonstrating how essential acetosyringone is to the transformation process.

### 3.3. Effect of Agar Concentration on Transformation Efficiency

**Figure 4:**
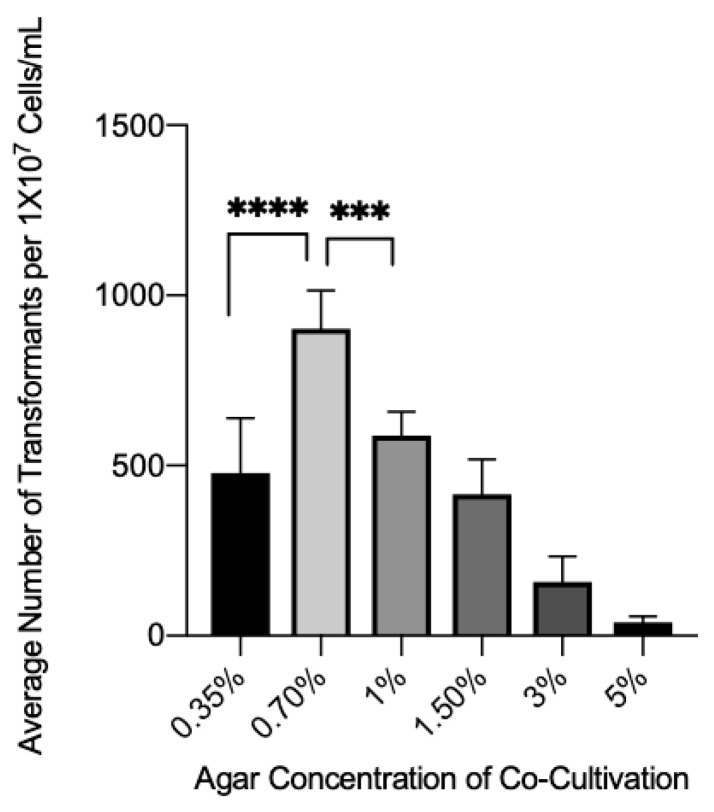
The effect of agar concentration in co-culture media on transformation efficiency. Wild-type *C. glabrata* and *A. tumefaciens* were mixed onto nitrocellulose membranes and plated onto co-culture media with different percentages of agar. Brackets with **** or *** above bars indicate groups with statistically significant differences. *** = *p* ≤ 0.001, **** = *p* ≤ 0.0001.

The influence of agar concentration in co-culture media was tested to observe what effect it might have on the transformation efficiency, since agar concentration had a striking effect on *Cryptococcus neoformans* transformation frequency [25]. Compared to the baseline concentration of 1.5%, decreasing the agar percentage to 0.7% showed a significant increase in the yield of the transformants (Figure 4). However, an increase in agar concentration showed a steady but significant decrease in the transformation efficiency. Interestingly, a ~30% increase or decrease in agar concentration from 0.70% led to a significant decrease in the number of transformants observed, demonstrating that there is a narrow range for optimum agar concentration.

### 3.4. Effect of the Bacterial:Fungal Cell Ratio on Transformation Efficiency

**Figure 5:**
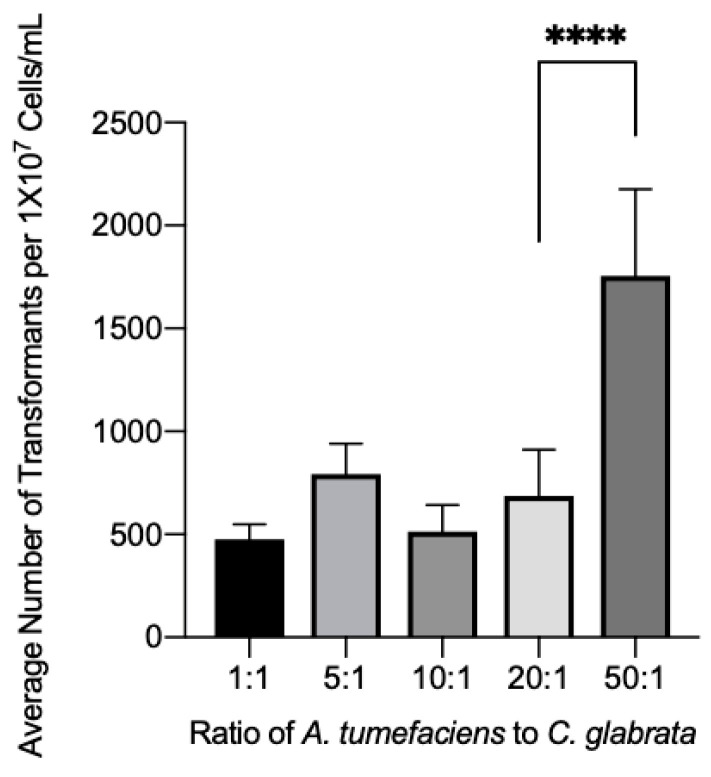
The influence of the ratio of *A. tumefaciens* cells to *C. glabrata* cells. One hundred microliters of wild-type C. glabrata were placed on nitrocellulose membranes with various amounts of *A. tumefaciens* to determine the best ratio of cells to achieve the highest transformant yield. The membranes with *C. glabrata* and *A. tumefaciens* were then placed on to co-culture media and incubated for 72 h at 26 °C. Brackets with **** above bars indicate groups with statistically significant differences. **** = *p* ≤ 0.0001.

Another crucial step in optimizing the ATMT system was to determine the best bacteria-to-fungal cell ratio. The baseline ratio of 10 *A. tumefaciens* cells to 1 yeast cell is frequently used in fungal transformation systems. We used this ratio as the starting point to determine the optimum ratio for *C. glabrata* transformation. The amount of *A. tumefaciens* cells then decreased or increased to observe the effect of different ratios (Figure 5). The ratios tested were 1:1, 5:1, 10:1, 20:1, and 50:1, to compare the possible differences in transformation efficiency. Ratios of 1:1, 5:1, and 20:1 showed no significant change in the transformant yield from the baseline ratio (10:1). However, a significant change in transformation efficiency was seen at the 50:1 ratio, which increased transformation 3-fold compared to the baseline ratio.

**Figure 6:**
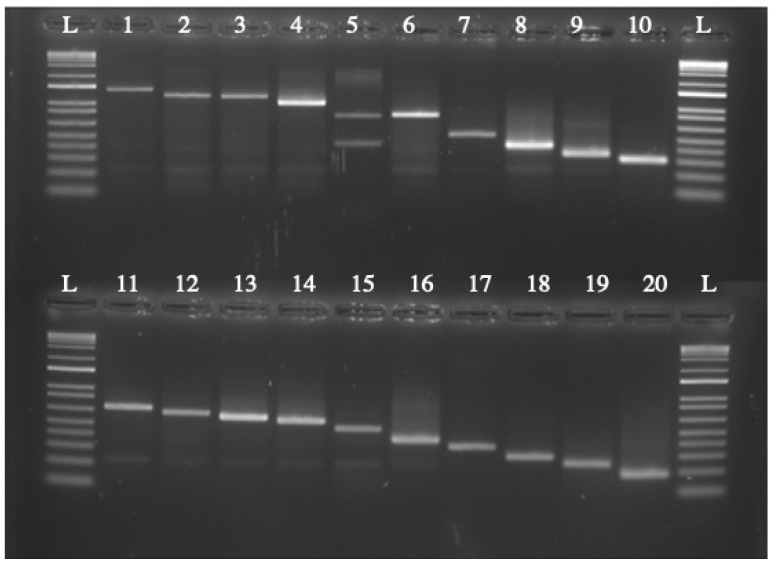
Identification of insertion site of 50:1 ratio of *A. tumefaciens* to *C. glabrata* transformants using vectorette PCR. (A). To confirm that increasing the ratio of *A. tumefaciens* to *C. glabrata* did not cause double insertions vectorette PCR was used to identify the insertion sites. One out of twenty samples showed a double insertion (Lane 5), meaning multiple insertions occurred at a 5% rate. The samples were flanked on each side with a 1.0 Kb Plus ladder size standard (L) (New England Biolabs, Ipswich, MA, USA).

While increasing the ratio of *A. tumefaciens* to *C. glabrata* cells showed a significant increase in transformation efficiency, there were concerns that increasing the bacteria to such a high level could lead to multiple insertions. To test for multiple insertions, 40 colonies were randomly selected from the 50:1 ratio transformation. Vectorette PCR was then used to identify the insertion site. Twenty insertions yielded vectorette PCR products, with only one double insertion identified, yielding a 5% rate of double insertions (Figure 6). Potential clonal insertions were screened by comparing band sizes after agarose gel electrophoresis, and any samples with similar-sized bands were sequenced. Sequencing of six of the 20 samples (3 pairs) showed one pair to be clonal, suggesting a 95% rate of unique transformants. Taken together, these results suggest that 90% of the transformants were single, unique insertions at the optimum bacterium:yeast ratio.

### 3.5. Optimized Transformation Protocol

**Figure 7:**
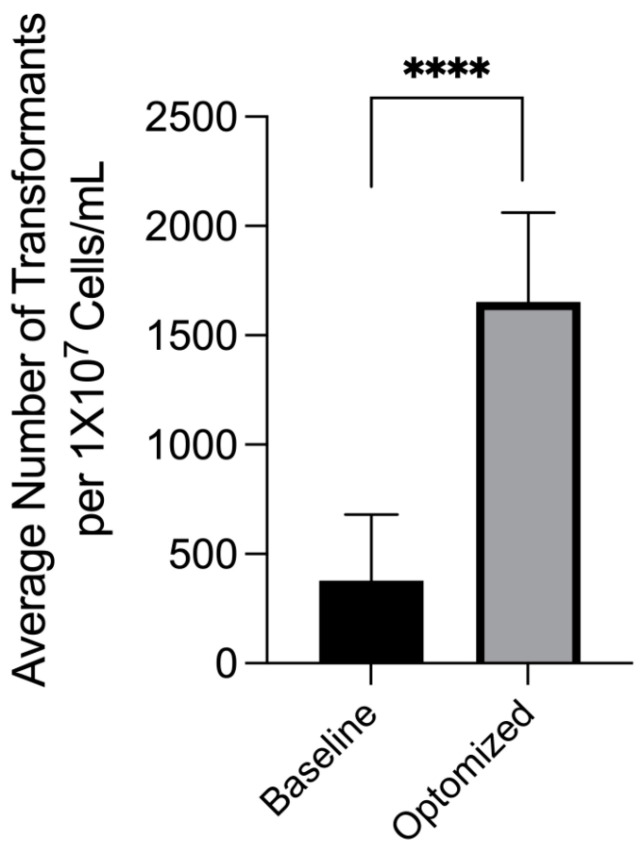
Comparison of optimized conditions to standard baseline conditions. Using the data above, optimal conditions were chosen and compared to the standard protocol. Transformation frequency improved roughly 4-fold. Brackets with **** above bars indicate groups with statistically significant differences. **** = *p* ≤ 0.0001.

To optimize transformation efficiency, we combined all the individual optimal conditions, which included optimized co-cultivation temperature, acetosyringone conditions, agar concentration, and bacteria/yeast cell ratio. The optimized protocol showed a significant difference in transformation frequency from the baseline group, which had a transformant yield of 378 transformants per 1 × 10^7^ cells/mL. The optimized conditions had an approximate 4-fold increase in average transformants (Figure 7).

## 4. Discussion

By optimizing these conditions, we significantly increased the transformation efficiency by 4-fold compared to that obtained using the standard transformation protocol. Under the optimized conditions, the ATMT system generated a 95% rate of unique transformants with minimal double insertions (5%) and minimal clonal redundancy (5%) for a total of 90% independent single insertion transformants. These percentages suggest an efficient transformation system, which is an important observation for insertional mutagenesis since double insertions can complicate the determination of gene function. Conversely, high numbers of clonal insertions reflect a lower unique transformant frequency, which can make screening more laborious due to the need to plate more transformants.

While many variables could be manipulated to yield increased transformation frequencies, not all worked. We found that zymolyase increased transformation frequency but not significantly. It is possible that more accurately titrating enzyme amounts or modifying spheroplasting conditions could significantly improve transformation frequency, but this improvement would come at the expense of making the protocol more laborious. Nevertheless, investigating cell wall integrity as a way to improve ATMT could be a fruitful area, particularly for hard-to-transform fungi. When we investigated cell wall damage in *C. neoformans* ATMT, we found that physical damage to the cell wall using bead beating increased the transformation frequency as beating time was increased, although exceeding a specific time point led to a large decrease in transformant number [25].

The variables that we studied are by no means complete. Other variables have been shown to enhance transformation in other fungal systems. For example, a study by Roberts et al. showed that blocking purine synthesis during the transformation process by adding mizoribine into the co-culture media led to a significant increase in the average number of *S. cerevisiae* transformants [26]; however, in our hands, the average transformant frequency of *C. glabrata* was not affected significantly. In *Paracoccidioides brasiliensis* mizoribine was found to have a negative effect on transformation frequency [27]. Another study done by Veluthambi et al. showed that introducing opines to *Agrobacterium* during the induction of *Vir* genes led to a significant increase in the average number of transformants in cotton root tips [24]. We did not see a significant improvement after the addition of an opine (Nopaline) to the *C. glabrata* transformation but instead found a strikingly negative effect on transformation frequency. Additional variables not tested during this study that could improve the transformation efficiency of *C. glabrata* include co-cultivation time, type of filter membrane (nitrocellulose, cellophane, and nylon) that the bacteria and yeast cells are incubated on, and different *A. tumefaciens* plasmid and strain combinations [27]. 

It is probably reasonable to conclude that there are certain common parameters that are necessary for fungal ATMT systems, and these should serve as a starting point for any transformation protocol. Incubation temperature, acetosyringone concentration, and perhaps even bacteria/fungus ratio likely have fairly narrow windows that can be manipulated and are fairly similar across fungal transformation systems. Other factors that appear to be fungus specific likely have much larger ranges for variation. The mizoribine and Nopaline results were striking, as they have shown significant positive effects on other fungi. Similarly, the agar concentration in the *C. glabrata* ATMT system was actually found to be opposite of what we have previously observed for *C. neoformans*. In the *C. neoformans* system, increasing concentrations of agar (up to 8%) increased transformation frequency tremendously, while decreasing concentrations in the *C. glabrata* system improved transformation frequency. Taken together, these observations suggest that once initial ATMT transformation parameters yield sufficient transformants, investigating each parameter one by one may be necessary to optimize the system, since different fungi may respond differently to different parameters. 

Once a high enough transformation frequency is obtained, ATMT can be used in different ways. While we did not use replica plating to screen for mutants, we did screen for pink colonies, which are typically *ADE1* or *ADE2* auxotrophs in yeast, and observed many pink colonies and confirmed that they required adenine for growth (data not shown). Just as important, we scaled up the procedure and were able to bank a library of 2 × 10^5^. Because we used a dominant drug resistance marker that is not part of the *C. glabrata* genome, transformants in the library should be at normal fitness levels, which could make this ATMT system useful for virulence studies. The regulatory elements (promoter and terminator) for the ATMT plasmid are heterologous to prevent biased integration at homologous *C. glabrata* sites, which could occur if we had used native *C. glabrata* regulatory elements to control the expression of the NAT gene. The promoter and terminator were derived from *C. albicans*; consequently, other markers could be used with the promoter and terminator in our system. It is not possible, however, to easily evict the NAT construct, since no repeated sequences were included in the plasmid that could function similar to the *C. albicans* SAT flipper or *hisG* repeat-mediated recycling strategies. Therefore, unless a new construct is developed, each transformed strain will be useful only for studying the effect of the disrupted gene. The vectorette strategy easily allows identification of the insertion site, so repeating the disruption using classical knockout and complementation approaches is possible.

In summary, we developed and optimized an ATMT system for *C. glabrata* and showed that co-cultivation temperatures between 24 °C to 26 °C with a bacteria to fungus ratio of 50:1, an acetosyringone concentration of 200 μL of 100 mM per 100 mL in both co-culture media and induction media, and an agar concentration in the co-cultivation medium of 0.7% yielded the maximum number of transformants. While the variables in our protocol are common to many fungal ATMT systems, we found that a given variable can be optimized for a specific species of fungus and then combined to yield a useful protocol. Depending on the specific application and how great the need is for maximum transformation, further optimization may be possible and needed. For *C. glabrata,* we found this protocol sufficient to perform routine transformations that could be used for experiments such as replica plate screening for specific mutants, or for recovering libraries of transformants suitable for banking. Taken together, this report is the first description of ATMT working in *C. glabrata*. It adds another important method to the tools that can be used to manipulate this fungus, which is becoming increasingly important as a pathogen.

## Data Availability

Not applicable.
